# Polygenic risk for schizophrenia and bipolar disorder in relation to cardiovascular biomarkers

**DOI:** 10.1007/s00406-023-01591-0

**Published:** 2023-05-05

**Authors:** Elina J. Reponen, Thor Ueland, Jaroslav Rokicki, Francesco Bettella, Monica Aas, Maren C. F. Werner, Ingrid Dieset, Nils E. Steen, Ole A. Andreassen, Martin Tesli

**Affiliations:** 1 NORMENT, Norwegian Centre for Mental Disorders Research, Oslo University Hospital and University of Oslo, Nydalen, P.O. Box 4956, N- 0424 Oslo, Norway; 2 Institute of Clinical Medicine, University of Oslo, Oslo, Norway; 3 Research Institute of Internal Medicine, Oslo University Hospital Rikshospitalet, Oslo, Norway; 4 K.G. Jebsen Thrombosis Research and Expertise Center, University of Tromsø, Tromsø, Norway; 5 Centre for Research and Education in Forensic Psychiatry, Department of Mental Health and Addiction, Oslo University Hospital, Oslo, Norway; 6 Department of Mental Health Research and Development, Division of Mental Health and Addiction, Vestre Viken Hospital Trust, Drammen, Norway; 7 Division of Mental Health and Addiction, Acute Psychiatric Department, Oslo University Hospital, Oslo, Norway; 8 Department of Mental Disorders, Norwegian Institute of Public Health, Oslo, Norway

**Keywords:** Polygenic risk, Bipolar disorder, Schizophrenia, Cardiovascular biomarkers, BMI

## Abstract

Individuals with schizophrenia and bipolar disorder are at an increased risk of cardiovascular disease (CVD), and a range of biomarkers related to CVD risk have been found to be abnormal in these patients. Common genetic factors are a putative underlying mechanism, alongside lifestyle factors and antipsychotic medication. However, the extent to which the altered CVD biomarkers are related to genetic factors involved in schizophrenia and bipolar disorder is unknown. In a sample including 699 patients with schizophrenia, 391 with bipolar disorder, and 822 healthy controls, we evaluated 8 CVD risk biomarkers, including BMI, and fasting plasma levels of CVD biomarkers from a subsample. Polygenic risk scores (PGRS) were obtained from genome-wide associations studies (GWAS) of schizophrenia and bipolar disorder from the Psychiatric Genomics Consortium. The CVD biomarkers were used as outcome variables in linear regression models including schizophrenia and bipolar disorder PGRS as predictors, age, sex, diagnostic category, batch and 10 principal components as covariates, controlling for multiple testing by Bonferroni correction for the number of independent tests. Bipolar disorder PGRS was significantly (*p* = 0.03) negatively associated with BMI after multiple testing correction, and schizophrenia PGRS was nominally negatively associated with BMI. There were no other significant associations between bipolar or schizophrenia PGRS, and other investigated CVD biomarkers. Despite a range of abnormal CVD risk biomarkers in psychotic disorders, we only found a significant negative association between bipolar disorder PGRS and BMI. This has previously been shown for schizophrenia PGRS and BMI, and warrants further exploration.

## Introduction

Psychotic disorders are associated with reduced life expectancy with cardiovascular diseases (CVD) as a major contributor: mortality rate ratios for CVD are 2–3 in individuals with schizophrenia (SCZ) and almost 2 in individuals with bipolar disorder (BD) compared with the general population [1]. Metabolic dysregulation in patients with psychotic disorders contributes to the elevated CVD risk with elevated body mass index (BMI), type 2 diabetes (TD2), and dyslipidemia occurring at a higher rate in this patient group compared with the general population[2]. Atherogenic lipid ratios are established predictors of CVD risk in the general population [3] and correlate well with intima media-thickness in arteries [4, 5], which is increased in SCZ and BD [6, 7]. We have recently demonstrated that 26–31% of patients with psychotic disorders with a mean age of 31 years, have elevated total cholesterol/high-density lipoprotein; HDL-c (TC/HDL) or triglyceride/HDL-c (TG/HDL) levels, respectively. Furthermore, above threshold levels of these lipid ratios were strongly correlated with increased plasma concentration of the vascular inflammatory markers myeloperoxidase (MPO) and C-reactive protein (CRP) as well as low concentration of the cardio-protective metabolic marker adiponectin [8, 9]. These proteins seem to be good CVD risk markers in populations with metabolic disturbances [10–12].

Environmental factors such as smoking, lack of preventive factors such as physical activity, and inadequate access to, or follow-up from health-care services, together with adverse metabolic effects of antipsychotic medication contribute to the elevated CVD risk associated with psychotic disorders. However, dysregulated metabolic factors have also been found in drug-naive individuals with SCZ or BD [13–15], suggesting that other underlying factors may have a greater influence on CVD risk than adverse metabolic effects of antipsychotic medication.

SCZ and BD are conditions with a multifactorial etiology and high heritability estimates (60–80%)[16]. Both disorders are highly polygenic, and there is robust evidence for cross-heritability between SCZ and BD [16, 17]. Further, several lines of evidence support the hypothesis of shared genetic factors between psychotic disorders and CVD risk [18–20], although the biological mechanisms are still elusive. A recent study suggested that individuals with SCZ might be genetically predisposed to several cardiovascular and metabolic abnormalities independent of medication side effects [20]. However, the extent to which the dysregulated CVD biomarkers across different biological pathways are explained by common genetic factors is still unknown.

Herein, we investigate this question using SCZ and BD polygenic risk scores (PGRS) in a naturalistic case–control cohort of patients with psychotic disorders and healthy controls. We selected established CVD biomarkers such as BMI, insulin resistance and atherogenic lipid ratios and well as inflammatory markers that are good CVD risk markers in populations with metabolic disturbances [10–12], and that we have shown to correlate with CVD risk in our patients [8, 9].

## Methods

### Design

The current cross-sectional study is a part of the ongoing Thematically Organized Psychosis (TOP) Study at the Norwegian Centre for Mental Disorders Research (NORMENT). The sample for this study consists of patients and healthy controls included from the year 2002 to 2017.

### Sample

#### Patients

Patients in the TOP study are recruited from hospitals and outpatient clinics in the Oslo, Trondheim, and Lillehammer regions in Norway. In this study, we included 699 patients with SCZ spectrum (541 fasting), and 391 with BD spectrum (331 fasting) with age between 18 and 65 years.

The diagnostic evaluation of the patients was based on the SCID-1 (Structured Clinical Interview in Diagnostic and Statistical Manual of Mental Disorders, 4^th^ Edition (DSM-IV) axis I Disorders). The inter-investigator diagnostic agreement has previously been evaluated to a satisfying level of 82%, with overall *ĸ* = 0.77 (CI 0.60–0.94)[21].

A diagnosis of SCZ spectrum included the diagnoses of schizophrenia, schizoaffective disorder, schizophreniform disorder, and psychotic disorder not otherwise specified, while a diagnosis of BD spectrum included the diagnoses of bipolar I, bipolar II, and bipolar disorder not otherwise specified.

#### Healthy controls

The current study included 822 healthy controls (HC), of which 165 were fasting. The HC were between 18 and 65 years old and randomly selected from statistical records (www.ssb.no) in the Oslo region. Exclusion criterion for HC was current or previous mental illness and/or serious mental illness in their family members, assessed with the clinical interview for severe mental traits, as well as the Primary Care Evaluation of Mental Disorders (PRIME MD).

#### Exclusion criteria related to the cardiovascular biomarkers

Since we evaluated a panel of CVD risk biomarkers that also included inflammatory markers, the exclusion criteria for all participants were signs of on-going infections, C-reactive protein (hs-CRP) > 20 mg/L, comorbid autoimmune or inflammatory diseases or cancer, and treatment with immune-modulating medication. All participants with insulin levels < 400 pmol/L were excluded for valid calculation of insulin resistance. A non-fasting status was an exclusion criterion for the biomarkers measured in plasma.

### Cardiovascular biomarkers

#### Selection criteria

We selected CVD risk markers representing the following partly overlapping pathways:

1. Body mass related markers: Body mass index (BMI), and fasting markers of adipose tissue inflammation; adiponectin and the leptin/adiponectin ratio (L/A).
2. Markers of dyslipidemia; fasting TC/HDL and TG/HDL ratio.
3. Markers of insulin resistance: homeostatic model assessment for insulin resistance (HOMA-IR)(i.e. based on fasting glucose and insulin)
4. Markers of vascular low-grade inflammation: fasting levels of CRP and MPO

##### BMI

All participants were weighed on calibrated digital scales under standard conditions (non-fasting), height was measured with standard methods and body mass index (BMI) calculated as the ratio between weight in kilograms (kg) and squared height in meters (m^2^). While the plasma markers were restricted to fasting samples, BMI calculations were available in 699 SCZ, 391 BD and 822 HC.

#### Blood samples

Blood samples for the cardiovascular biomarkers were collected fasting, and for most of the participants performed between 8 and 11 am. Blood samples were drawn into EDTA tubes, stored at room-temperature for 45 min and placed in a refrigerator at 4 °C. They were then transported to the Biobank the following workday, where 2 × 9 ml EDTA tubes were centrifuged at 1800 g for 15 min. Plasma was collected and stored at -80 °C in multiple aliquots [22]

#### Measurement of biomarkers

##### Lipids

Plasma levels of total cholesterol (TC), triglyceride (TG), and high-density lipoprotein (HDL-c) were measured on an Integra 800 instrument from Roche Diagnostics, according to standard methods. Atherogenic lipid ratios TC/HDL and TG/HDL were then calculated by us using standard entities.

##### Inflammatory markers

Plasma levels of MPO, CRP, leptin, and adiponectin were analyzed as previously described [8, 9]. Leptin/adiponectin ratio was calculated by us using standard entities.

##### Insulin resistance

Plasma levels of glucose and insulin were analyzed at the Department of Medical Biochemistry, Oslo University Hospital. Glucose levels were analyzed using standardized platforms from Roche Diagnostics. Insulin was analyzed at the Hormone Laboratory by radioimmunoassay (RIA) using standard methods. We estimated insulin resistance using the Homeostasis Model Assessment for Insulin Resistance (HOMA-IR) [23].

##### Polygenic risk scores

DNA was extracted from the collected blood samples. Genotyping was performed on Human Omni Express-24 v.1.1 (Illumina Inc., San Diego, CA, USA) at deCODE Genetics (Reykjavik, Iceland). Quality control was performed using PLINK 1.9 [24].

Briefly, variants were excluded if they had low coverage (< 95%), had low minor allele frequency (MAF) (< 0.01), deviated from Hardy–Weinberg equilibrium (*p* < 10^−4^), or occurred at significantly different frequencies in different genotyping batches (FDR < 0.5). Whole individual genotypes were excluded if they had low coverage (< 95%) or high likelihood of contamination (heterozygosity above mean + 5 standard deviations). MaCH [25] was used to impute the genotypes of all participants onto reference haplotypes derived from samples of European ancestry in the 1000 Genome Project (genomic build GRCh37). The polygenic risk score for SCZ (SCZ PGRS) and for BD (BD PGRS) were based on the latest meta-analyses of SCZ and BD from the Psychiatric Genomics Consortium [26, 27] after exclusion of individuals included in the current study. The summary statistics were quality controlled by removing variants that met any of the following conditions: MAF < 0.05; imputation quality (ratio between observed and expected allelic variance) < 0.8; not present in more than half of the sub studies. Variants from the MHC region were also excluded. The remaining variants were clumped into independent regions on the basis of the linkage disequilibrium structure of the 1000 Genomes Phase III European population. PLINK v1.9 was used with the following parameters: –clump-p1 1.0 –clump-p2 1.0 –clump-r2 0.2 –clump-kb 500. The allelic dosage coefficients (or logarithms of the odds ratios) of the variants with minimum p-values from all independent regions were used in constructing the SCZ PGRS and BD PGRS. These were calculated for all individuals following Purcell et al.'s [28] recipe of multiplying the number of effect alleles they carried by the allelic dosage coefficients calculated in the meta-analysis. Only European subjects were included in our sample to avoid confounding from population stratification. To investigate the clustering of alleles due to ancestry/population stratification a principal component analysis was conducted using PLINK [24] on a set of independent variants. This yielded 20 genetic principal components, and the 10 first of these were selected for further analyses. These genotyping and imputation procedures have previously been described in detail [29].

### Statistical analyses

All the statistical analyses were performed with the statistical software package R (http://www.r-pro- ject.org/). An ANCOVA model (sex and age as covariates) was applied to determine the differences in the 8 CVD biomarkers between SCZ (*n* = 541), BD (*n* = 331), and HC (*n* = 165), with post hoc Tukey’s test comparing groups pairwise.

CVD biomarkers were used as outcome variables in linear regression models including SCZ and BD PGRS as predictor variables, and diagnosis (BD, SCZ), sex, age, batch, and the 10 first principal components as covariates. Among the PGRS thresholds (*P* < 0.01, *P* < 0.05, *P* < 0.1, *P* < 0.5, *P* < 1), we focused primarily on the *P* < 0.05 PGRS threshold, which had the highest predictive value of SCZ and BD in the current sample.

We finally investigated the correlation between BMI and SCZ PGRS and BD PGRS in a larger sample available for these variables. An ANCOVA model was applied to determine the differences in BMI between SCZ spectrum cases (*n* = 699), BD spectrum cases (*n* = 391), and HC (*n* = 822), with post hoc regression models comparing groups pairwise. BMI was used as the outcome variable in linear regression models including SCZ PGRS and BD PGRS as predictor variables, and diagnosis (BD, SCZ), sex, age, batch, and the 10 first principal components as covariates.

To account for multiple comparisons, we first clustered data to calculate the number of effective independent tests needed. To determine the optimal number of clusters we used NbClust function CITE1 with majority vote on 30 different indices using Euclidean distance and ward. D2 clustering method described in detail at https://www.jstatsoft.org/v61/i06/. 7 indices suggested 2 clusters and 7 times 3 clusters were proposed. We have chosen 3 as a more conservative estimation of effective independent tests needed times 2 disorders. Therefore, all p-values are Bonferroni corrected for 6 multiple comparisons.

## Results

As presented in Table 1, the clinical characteristics of the study population are as follows: patients with BD were significantly older compared both with HC and patients with SCZ. BD patients were more frequently of female sex than HC, and HC more frequently so than SCZ. The patients in general had significantly higher BMI, and SCZ patients had higher BMI than BD, and BD had higher BMI than HC.

**Table 1:** Demographics and distributions of cardiovascular biomarkers and polygenic risk scores (PGRS) across the three diagnostic groups

Demographics	SCZ mean (sd)	BD mean (sd)	HC mean (sd)	*F*	*P* value	Pairwise comparison
Age	30.6 (9.7)	33.9 (11.9)	33.3 (9.4)	25.02	1.75 × 10^–11^	SCZ < BD, HC
Sex, N females	394/960 (41%)	309/506 (61%)	468/1018 (46%)	27.7	1.27 × 10^–12^	BD > HC > SCZ
Cardiovascular biomarkers^1^						
BMI	26.27 (5.16)	25.84 (4.7)	24.57 (3.76)	43.214	< 2 × 10^–16^	SCZ > BD > HC
CRP	2.4 (2.83)	2.18 (2.75)	1.65 (2.27)	5.485	0.00425	SCZ > BD, HC
MPO	426.57 (436.51)	467.82 (479.65)	231.39 (297.38)	6.156	0.002227	SCZ, BD > HC
Adiponectin	11.46 (5.95)	12.16 (5.79)	10.91 (4.91)	3.784	0.023	SCZ, BD, HC
Leptin/adiponectin ratio	112.06 (191.95)	90.05 (92.76)	63.09 (56.23)	8.004	0.000352	SCZ > BD > HC
Total cholesterol/HDL	4.17 (1.62)	3.74 (1.4)	3.43 (1.17)	25.49	1.41 × 10^–11^	SCZ > BD > HC
Triglycerides/HDL	1.3 (1.28)	1.08 (1.18)	0.84 (0.83)	12.264	5.31 × 10^–6^	SCZ, BD > HC
HOMA2_IR	1.75 (1.04)	1.49 (0.78)	1.23 (0.67)	26.537	5.19 × 10^–12^	SCZ > BD > HC
Polygenic risk scores^2^						
SCZ PGRS (0.05)	0.32 (1.04)	0.00 (0.92)	-0.27 (0.91)	37.256	< 2 × 10^–16^	SCZ > BD > HC
BD PGRS (0.05)	0.19 (1.01)	0.12 (1.01)	-0.22 (0.95)	89.022	< 2 × 10^–16^	SCZ, BD > HC

*BMI* Body mass index, *CRP* C-reactive protein, *MPO* myeloperoxidase, *HDL* High density lipoprotein, *HOMA2-IR* homeostatic model assessment for insulin resistance, *SCZ* Schizophrenia, *BD* Bipolar disorder

^1^Corrected for age, and sex

^2^Normalized and corrected for age, sex, batch and 10 first principal components

For the plasma biomarkers, the results have been previously reported in these patients with some minor differences with regard to number of patients and HC [8, 9]. As shown in Table 1, the leptin/adiponectin and TC/HDL ratios as well as HOMA-IR was higher in SCZ compared to BD and was higher in BD compared to HC. Further, the TG/HDL ratio and MPO was higher in SCZ and BD compared to HC while CRP was higher in SCZ compared to BD and HC.

As presented in Table 1, the SCZ PGRS was highly significantly associated with SCZ, and BD PGRS was significantly associated with both BD and SCZ.

**Fig. 1:**
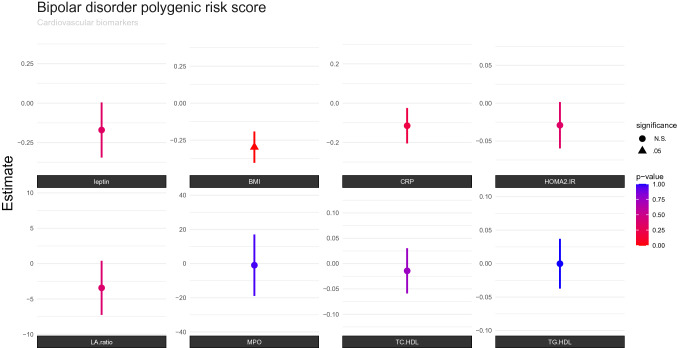
Associations between cardiovascular biomarkers and bipolar disorder polygenic risk score (PGRS), after correction for sex, age, diagnosis, batch and 10 first principal components. The *P* value (0.05) is Bonferroni corrected for the number of independent tests, and the threshold is marked with colour codes according to statistic significance level

**Fig. 2:**
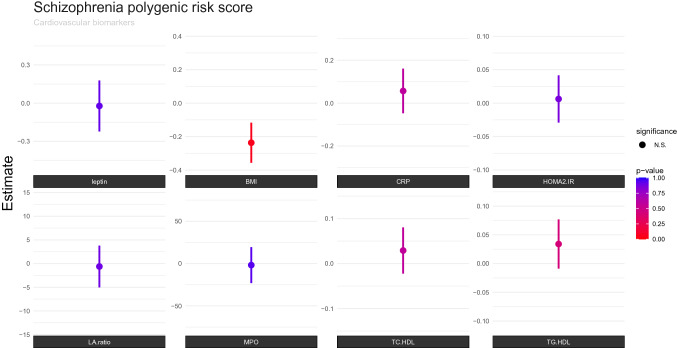
Associations between cardiovascular biomarkers and schizophrenia polygenic risk score (PGRS), after correction for sex, age, diagnosis, batch and 10 first principal components. The *P* value (0.05) is Bonferroni corrected for the number of independent tests, and the threshold is marked with color codes according to statistic significance level

The results of the final linear regression model are presented in Table 2 as well as in Figs. 1 and 2. Of the eight CVD biomarkers, only BMI was found to be significantly negatively associated with BD PGRS. The corrected p-value for the BD PGRS was (*p* = 0.0297). BMI was also found nominally negatively associated with SCZ PGRS (uncorrected *p* = 0.0483), although did not remain significant (*p* = 0.2900) after correction for multiple comparisons.

**Table 2:** Associations between cardiovascular biomarkers and polygenic risk scores (PGRS) for bipolar disorder (BD) and schizophrenia (SCZ)

	SCZ PGRS (*P* = 0.05 threshold)			BD PGRS (*P* = 0.05 threshold)		
Cardiovascular biomarkers	*P* value	Coefficient	*P* corrected	*P* value	Coefficient	*P* corrected
BMI	0.0483	− 0.237	0.290	0.00495	− 0.297	0.0297
CRP	0.59	0.057	1	0.20	− 0.115	1
MPO	0.93	− 1.775	1	0.96	− 0.946	1
Adiponectin	0.91	− 0.022	1	0.33	− 0.170	1
Leptin/adiponectin ratio	0.89	− 0.614	1	0.37	− 3.436	1
Total cholesterol/HDL	0.57	0.029	1	0.75	− 0.014	1
Triglyserids/HDL	0.43	0.034	1	1.00	0.000	1
HOMA2_IR	0.86	0.006	1	0.34	− 0.029	1

*BMI* Body mass index, *CRP* C-reactive protein, *MPO* myeloperoxidase, *HDL* High density lipoprotein, *HOMA2-IR* homeostatic model assessment for insulin resistance

^1^Corrected for age, sex, batch and 10 first principal components

## Discussion

We investigated whether CVD biomarkers were explained by common genetic factors using SCZ and BD PGRS in a naturalistic sample of patients with SCZ and BD and healthy controls. To our knowledge, this is the first naturalistic study demonstrating this inverse association between BD PGRS and BMI, which survived multiple correction testing. An inverse association has recently been demonstrated between SCZ PGRS and BMI [30]. We also herein found a nominal negative association between SCZ PGRS and BMI, which did not remain significant after correction for multiple testing. There were no other nominally significant associations between BD or SCZ PGRS and any of the other CVD biomarkers.

Our findings concurrently support the genetic overlap between BD and SCZ, previously demonstrated in twin studies [31], and molecular genetic studies [17, 32]. BD and SCZ share several clinical characteristics [33], and similar somatic comorbidities [34]. Overweight and obesity are frequently observed both in individuals with BD and with SCZ, and elevated BMI has been shown to be associated with antipsychotic medication, and with behavioral and environmental risk factors [2]. The pathophysiological mechanisms that could explain the inverse genetic association between BMI, and BD as we demonstrate here, and for SCZ previously [18], are unknown. Epidemiological studies have shown that low BMI is a risk factor for SCZ [35], and it has been suggested that poor nutritional status at an early age may affect neural development, leading to SCZ [36]. For BD, the epidemiological, clinical and previous genetic studies have shown positive correlations between BMI and BD. However, eating disorders such as anorexia nervosa, bulimia nervosa, or binge eating disorder occur at high rate in BD, resulting often in weight loss. It has been reported that between 5.3% and 31% of patients with BD suffer from a eating disorder [37], and a genetic overlap has been found between these disorders [38]. Additionally, it has been suggested that the active phase symptoms in psychotic disorders (such as emotion dysregulation and affect liability) [39] contribute to development of eating disorders and poor nutritional status. Psychotic symptoms have also been shown to occur transiently in the course of an eating disorders [40]. Further, lower genetic disposition to elevated BMI in BD has been shown in those BD patients with psychosis, than those without psychotic symptoms [41]. As the pathophysiological mechanisms involved in the genetic association between BD and low BMI are still unknown, this matter warrants further exploration with different genetic methods and study designs.

GWAS analyses have shown a relation between SCZ and BD and CVD biomarkers, but the results have been somewhat inconsistent when comparing results of the different studies with a variation in methodologies and sample sizes [42, 43]. While recent studies from ours [44] and other groups [20] demonstrated extensive polygenic overlap between SCZ and BD and CVD phenotypes and traits, fewer have reported associations with dysregulated plasma levels of CVD risk markers, although associations with insulin resistance [45] and CRP levels have been detected [42]. Furthermore, the loci identified by these studies reveal mixed effect directions suggesting that genetic susceptibility for CVD may vary widely across subgroups of patients. Nonetheless, the lack of findings of positive direction of correlations in our study may underscore the importance of environmental and lifestyle factors on CVD risk in these patients.

### Limitations

Since fasting status effects the plasma levels of several of the investigated cardiovascular biomarkers, these were only analyzed in a subsample. This included the lipid parameters, inflammatory markers and insulin resistance, limiting the statistical power of this set of statistical analyses, and increasing the risk of type II errors. For the HC in our study environmental factors such as smoking status is not available, and were not included in the analysis.

## Conclusion

Our main finding was a negative correlation between BMI and polygenic risk for BD, and nominally significant correlation with polygenic risk of SCZ. There were no significant associations between polygenic risk of SCZ and BD and other biomarkers, such as lipid parameters, inflammatory markers, and insulin resistance. Our findings suggest that common genetic factors explain a limited proportion of the increased CVD risk in these disorders, and suggest that CVD prevention should focus on environmental and lifestyle factors, and improving health care services, as well as pharmacological treatment.

## Data Availability

The datasets presented in this article are not readily available because sharing of data to external parties has not been approved by the ethics committee. Requests to access the datasets should be directed to e.j.reponen@medisin.uio.no.
